# Comparison of spatial scan statistic and spatial filtering in estimating low birth weight clusters

**DOI:** 10.1186/1476-072X-4-19

**Published:** 2005-08-02

**Authors:** Esra Ozdenerol, Bryan L Williams, Su Young Kang, Melina S Magsumbol

**Affiliations:** 1 Department of Earth Sciences, 236 Johnson Hall, University of Memphis, Tennessee, 38152, USA; 2 Department of Preventive Medicine, University of Tennessee Health Science Center, Memphis, Tennessee, USA

## Abstract

**Background:**

The purpose of this study is to examine the spatial and population (e.g., socio-economic) characteristics of low birthweight using two different cluster estimation techniques. We compared the results of Kulldorff's Spatial Scan Statistic with the results of Rushton's Spatial filtering technique across increasing sizes of spatial filters (circle). We were able to demonstrate that varying approaches exist to explore spatial variation in patterns of low birth weight.

**Results:**

Spatial filtering results did not show any particular area that was not statistically significant based on SaTScan. The high rates, which remain as the filter size increases to 0.4, 0.5 to 0.6 miles, respectively, indicate that these differences are less likely due to chance. The maternal characteristics of births within clusters differed considerably between the two methods. Progressively larger Spatial filters removed local spatial variability, which eventually produced an approximate uniform pattern of low birth weight.

**Conclusion:**

SaTScan and Spatial filtering cluster estimation methods produced noticeably different results from the same individual level birth data. SaTScan clusters are likely to differ from Spatial filtering clusters in terms of population characteristics and geographic area within clusters. Using the two methods in conjunction could provide more detail about the population and spatial features contained with each type of cluster.

## Background

Spatial analysis of birth related morbidity (e.g., low birthweight) provides a way to identify inadequate health care access as well as potential environmental and behavioral causal factors. Cluster analysis is frequently used to identify an unusually high occurrence of morbidity that is clustered in space and time [1]. The purpose of this study is to examine the spatial characteristics of low birthweight using two distinct cluster analysis techniques and to compare the resultant clusters in terms of their socio-economic characteristics [2,3]. Both Spatial Scan Statistic (SaTScan) and Spatial filtering techniques are designed to overcome limitations in presenting data in an aggregated form where spatial patterns of low birth weight may vary in relation to the level of geographic area used.

This study will assess the spatial characteristics of infant birth weight throughout Shelby County, Tennessee from 2000 to 2002 with the use of Kulldorff's Spatial Scan Statistics or SaTScan and Rushton's Spatial filtering methods. Comparing the results of the two methods using the same input gives us more insight in to the spatial distribution of birth morbidity data. This is particularly relevant since there is little or no evidence of comparison of these two methods in the literature. The research questions are as follows: (1) Are low birthweight births clustered significantly in relation to maternal residence from 2000–2002 in Shelby County? (2) To what extent will the total area within SaTScan clusters differ from the total area within Spatial filtering clusters? (3) To what extent will the maternal and familial characteristics of those births within SaTScan clusters differ from those within Spatial filtering clusters? To assess both methods, we used individual point data by address-matching the latitude-longitude coordinates of the maternal residences at the time of delivery and assigning these to specific grid locations.

This case study demonstrates how the two methods better reflect spatial variation when individual point data is used. A methodological comparison can provide insight about the limitations and benefits of varying approaches when mapping morbidity. Especially since methodological studies of these techniques are scarce. This study also demonstrates a strategy for dealing with the issue of geographical scale, which is central to this type of small-area ecological study [4]. Information obtained from this study provides a useful foundation for prospective environmental health tracking. By comparing the results across different spatial scales, we hope to derive more reliable information on an important health concern in this region.

### Cluster analysis of low birth weight

Studies have examined the spatial characteristics of low birth weight and other health outcomes in relation to contaminant exposures using geographic information systems (GIS) [5-7]. Results can vary significantly depending upon the level of geographic scale that is analyzed [8-10]. For example, spatial patterns of birth morbidity vary in relation to area-based census tract, block group, and zip code level measures of socio-economic status [12]. Additionally, aggregation bias represents an inherent source of error for these analyses [13]. Aggregation bias results from the rather arbitrary means by which GIS aggregate individual cases at some geographic organizational unit such as a census tract. The coarser the spatial scale the higher the potential for aggregation bias. SaTScan and Spatial filtering techniques differ in how each aggregates individual level data. Consequently, aggregation bias may manifest itself distinctively in one method versus the other.

Using GIS to study the spatial characteristics of low birthweight is complicated by the lack of methodological guidance and the scarcity of accurate integrated spatial-morbidity databases [14-18]. A representative, but not exhaustive, set of methods exist for analyzing spatial clusters of birth morbidity with individual point data or aggregated data that still maintains the stability of the estimated rates by constructing a continuous smoothed map [19-21]. SaTScan and Spatial filtering techniques are the most commonly used spatial analysis methods in epidemiological research. The SaTScan method has been applied much more broadly and more frequently than the Spatial filtering approach.

### Spatial filtering process

The Spatial filtering methodology employs non-parametric statistical techniques as a tool in exploratory spatial data analysis. This method has been used to study clusters of congenital anomalies, infant mortality, and other forms of birth morbidity [3,18,20]. It works well with both aggregated data and individual point data. The estimated rate at a particular grid point can be defined as the observed rate within a fixed distance from the grid point. Once estimated rates are assigned to each grid point, isarithmic maps can be constructed in GIS. We assume that the probability of a case resulting to a low birthweight birth is equal to the proportion of all births in the region that resulted in low birthweight [3]. A 'smoothed' probability map can be drawn where the significance levels of high rates of low birth weight by percentage for each individual circle is calculated and mapped in isarithmic form.

### SaTScan process

The SaTScan methods has been used to study clusters of cancer morbidity and mortality, sudden infant death syndrome, congenital anomalies, and infectious diseases [2,4,5]. SaTScan estimates the probability that the frequency of events per trial at each vertex surpasses that expected by chance. It then creates an isoplethic map that shows the estimated probabilities. SaTScan uses circles and a non-parametric test statistic. It takes into account the observed number of low birth weight births inside and outside the circle when calculating the highest likelihood for each circle. SaTScan uses a circular window of different sizes that scans the study area until a certain percent (e.g., half) of the total population is included. This circle is the most probable cluster, and has a rate that is the least likely to happen by chance alone. SaTScan also accounts for multiple testing through the calculation of the highest likelihood of occurrence for all possible cluster locations and sizes [2,5,21]. Although a range of probabilities can be displayed using SaTScan, only the most highly significant estimates are displayed in this paper.

As mentioned earlier, SaTScan accounts for multiple testing and only conducts one test for the whole collection of window location and sizes [2,5]. It tests the null hypothesis against the alternative hypothesis that there is an elevated rate of low birth weight within the windows as compared to the outside. The method uses the likelihood ratio λ as the test statistic [22]. The significance of the test statistic λ is determined by a large number of replications of the data set generated under the null hypothesis in a Monte Carlo simulation. The likelihood ratio λ for each replica is computed, and the result is significant at the 0.05 level if the λ value of the real data set is among the top 5% of all the values, including the replicas. Secondary clusters with lower significance can also be identified. SaTScan generates an ASCII output file, which contains the log likelihood ratios and their significance levels for the census areas. In this study, the output file was imported into Arc GIS 9 to create cluster maps to visually examine and compare the clusters.

## Methodology

We conducted a retrospective ecological study of birth weight in Shelby County, Tennessee that included births from the years 2000 to 2002. Birth data was obtained from fixed-width electronic birth certificate files from the Tennessee Department of Health. Demographic data was obtained from the 2000 U.S. Census, Summary Files 1 and 3 [23]. Using the Arc GIS 9 software, the mothers' addresses were automatically matched with a digital street file. The digital street network used in this study was the Environmental System Research Institute (ESRI) street map, which was derived from the 2000 Census Topologically Integrated Geographic Encoding and Referencing system (TIGER) files [23]. The location of each address is shown in this example only as a generalized location in order to preserve the confidentiality of the individual records. Personal information was never identified or displayed in this study.

Birth weights of less than 2,500 g were defined as a low birth weight. There were a total of 42,394 births, including 4,794 low birth weight infants – comprising 11.30 % of the total births in Shelby County during the years of 2000 till 2002. After completing the address matching, each birth record was characterized by unique latitude and longitude location coordinates. We first analyzed these birth records as point patterns in their own right by applying the Spatial filtering technique and compared the results to SaTScan clusters.

### Spatial filtering method

By address matching birth records to a digital road map, we were able to compute low birth weight rates for each location on a grid, which covers the entire Shelby county area at approximately 0.4 mile intervals. In order to make any general conclusions about the results of Spatial filtering, we used multiple filter sizes such as 0.4 mile, 0.5 and 0.6 miles. We conducted a sensitivity analysis using differing Spatial filter sizes and the 0.4 size appeared to provide the optimal distance for this study. Progressively larger Spatial filtering of data removes local spatial variability, which eventually produces an approximate uniform pattern of low birth weight. We did not aggregate cases to the geometric or geographic centroid of the administrative units such as zip codes, and census tracts. Given the approximate 0.4 mile distance interval between grid intersections, there were 5,928 grid points in Shelby County. Meaningful low birth weight rates were estimated for 841 grid points that had at least 40 births within their 0.4 mile vicinity. The Spatial filter area surrounding each of these points is the area from which an estimate of the low birth weight rate is made. We counted the normal births and low birth weights within the area and assigned the observed rate to the location. When we repeated this for a grid of such estimates, we could interpolate the low birth weight rate as a continuous spatial distribution. Neighbouring grid points share circular patterns that overlap, thereby sharing observations. Isarithmic maps with a constant range of values were constructed in GIS after the estimated rates were assigned to grid points.

For each birth location, we generated a random number from a uniform distribution in the range of 1 through 1,000. For each of the 841 grid point locations, 1,000 Monte Carlo simulations were made and the 1,000 different low birth weight rates were rank-ordered. The percent of the simulated rates at each grid location that were less than the observed rate for the same grid location were computed and the levels of statistical significance were portrayed as isolines. Because testing the rates against 1,000 simulations is a form of exploratory spatial analysis, methods of representing the results are discretionary, and the investigator can adjust the results based on level of significance. For example, the isolines representing the significance levels of 80 %, 85 %, 90 % and 95 % could be color-coded. Additionally, the isolines are overlaid on the significant SaTScan clusters for the purpose of comparison.

These probabilities, portrayed as isarithmic maps, show areas that have significantly high rates of low birth weight. The isarithmic maps have many advantages in comparison with other conventional thematic maps that provide an indication of the level of a disease by area. They are not constrained by the borders of geographic units, and sudden transitions between levels of two neighbouring areas are avoided [24]. We used the inverse-distance weighing interpolation technique in constructing the isarithmic maps. Since inverse distance weighing represents the average of the values of the surrounding points, weighed by the inverse of the distance to those points, the process is based on the assumption of positive spatial autocorrelation depicting a continuous gradient exists between points in a linear way [24].

### SaTScan method

In our second method, we applied the SaTScan method developed by Kulldorff to detect local clusters [22]. We compared the smoothed maps with the data derived through SaTScan in order to determine whether areas with statistically significant rates were retained or smoothed out. The spatial level of data and time period used is the same one used previously in the Spatial filtering method. Since both methods perform better on the point data, we did not include any analyses of aggregated data. We chose the Bernoulli model, which required information about the location of a set of cases and controls. We selected low birth weight geocoded points as cases and normal birth weight geocoded points as the controls. We employed non-overlapping grid buffers spaced at 0.4 mile intervals and maximum spatial cluster sizes of 0.4 mile, 0.5 and 0.6 mile respectively just like in the previous method.

## Results

### Significant clusters by method

**Figure 1:**
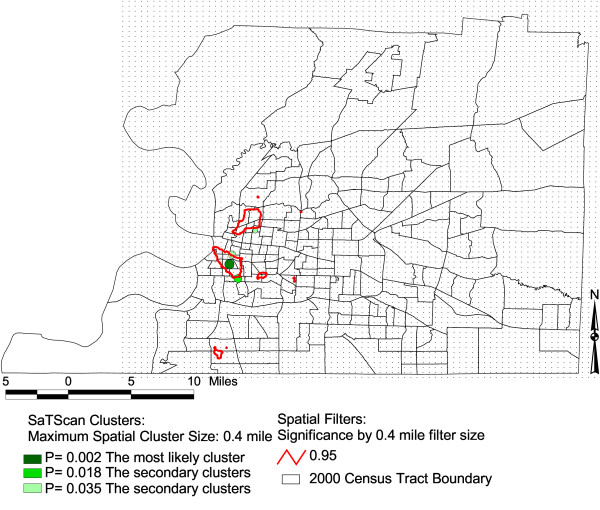
**Areas with statistically significant high rates of low birth weights, Shelby, TN, 2000–2002**. The maps show SaTScan clusters with a maximum spatial cluster size of 0.4 miles. It also shows significant Spatial filter clusters with a maximum 0.4 mile filter size.

**Figure 2:**
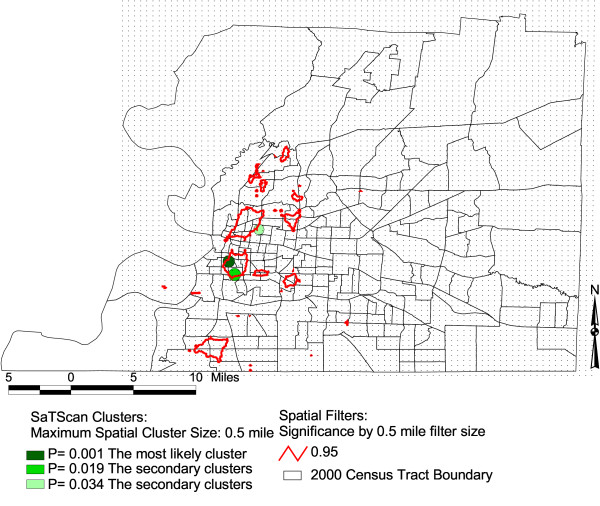
**Areas with statistically significant high rates of low birth weights, Shelby, TN, 2000–2002**. The maps show SaTScan clusters with a maximum spatial cluster size of 0.5 miles. It also shows significant Spatial filter clusters with a maximum 0.5 mile filter size.

**Figure 3:**
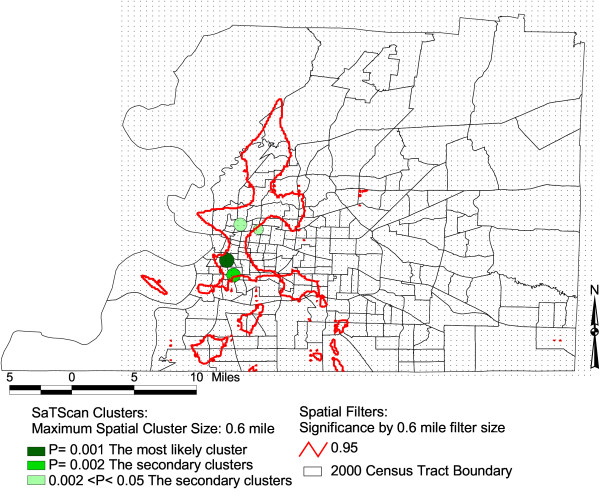
**Areas with statistically significant high rates of low birth weights, Shelby, TN, 2000–2002**. The maps show SaTScan clusters with a maximum spatial cluster size of 0.6 miles. It also shows significant Spatial filter clusters with a maximum 0.6 mile filter size.

In Figure 1, 2, and 3, we display the isolines of 95 % level Spatial filtering clusters using 0.4, 0.5 and 0.6 miles filter sizes for lump sum years of 2000–2002. These are overlaid on the most likely significant clusters of SaTScan for the purpose of comparison. We evaluated the effect of changes in filter size by creating maps with different filter sizes. The high rates of low birth weight remained as we increased the filter sizes to 0.4, 0.5 to 0.6 miles. This indicates that these differences are less likely due to chance. The 0.4 mile filter size or smaller filter sizes showed the local variability much better than the larger filter sizes of 0.5 and 0.6 miles (Figure 1). The 0.4 mile filter size resulted to five clustered areas. On the other hand, clusters increased in size and additional clusters emerged towards the northeastern part of the county when we used the 0.5 mile filter size (Figure 2). As we increased the filter size to 0.6 mile, the localized clusters unioned to a larger uniform pattern covering the western portion of the county (Figure 3). Table 1 shows the total area of the clusters for both methods with varying filter sizes. Once we applied a larger spatial filter size as 0.8 mile, the Spatial filtering technique lost the ability to detect elevated rates except for the most densely populated areas of Memphis.

**Table 1:** Area of clusters by method

Methods	Filtering Sizes or Maximum Spatial Cluster Sizes (miles)	Significance	Total Area of Shelby County (sq. meter)	Clustered Area (sq. meter)	% of Total Shelby County
Spatial filtering	0.40	≥ 0.95 level		6,676,687	0.32%
	0.50	≥ 0.95 level		27,191,960	1.32%
	0.60	≥ 0.95 level		119,492,954	5.80%
		
SaTScan	0.40	P1 (most likely) = 0.002		935,139	0.05%
		P2 (secondary) < 0.05	2,061,075,094	1,523,635	0.07%
	0.50	P1 (most likely) = 0.001		1,500,352	0.07%
		P2 (secondary) < 0.05		2,828,068	0.14%
	0.60	P1 (most likely) = 0.001		2,344,277	0.11%
		P2 (secondary) < 0.05		5,145,388	0.25%

On the other hand, SaTScan gave consistent results with larger filter sizes. The areas that have statistically significant rates adjusted after multiple testing also showed up as high rate areas on the Spatial filtering smoothed maps of low birth weight rates. SaTScan clusters were discrete compared to the continuous distribution of Spatial filtering clusters. The most likely SaTScan clusters and the continuous Spatial filtering clusters were concentrated in the western portion of Shelby County. Secondary clusters with less significance emerged as the cluster size increased. The resultant clusters were circular in shape within the predefined maximum spatial cluster size. The most likely clusters appeared in the same locations with larger radii with the exception of new secondary clusters in the vicinity.

Table 2 illustrates maternal and familial characteristics by cluster estimation method and type. The ethnic, economic, and educational characteristics of mothers whose births are in the cluster are quite similar irrespective of filter size. With both methods, the clusters become increasingly heterogeneous as the filter size increases. Although one would expect more heterogeneity as the sample size increases, it is possible that outer portions of the cluster start to encroach upon better educated and more affluent communities. The only major difference between the two methods was the total number of births within each respective type of cluster. Spatial filtering clusters include almost 3 times as many total births than does SaTScan at the 0.4 mile filter size. This may due in part to the fact that the total area covered by Spatial filtering clusters is almost 3 times greater than the total area covered by SaTScan clusters.

**Table 2:** Maternal and familial characteristics by cluster estimation method and type

Methods	Filtering Sizes or Maximum Spatial Cluster Sizes (miles)	Significance	Total Births within Cluster	Maternal Ethnicity within Cluster			Average Percentage of Mothers having Some College Education within Cluster	Average Percentage of Families Below Poverty level
						
				Caucasian	African American	Others		
Spatial filtering	0.40	≥ 0.95% level	1,250	1.4%	98.3%	0.2%	13.9%	48.7%
	0.50	≥ 0.95% level	2,509	2.1%	97.6%	0.3%	16.2%	40.6%
	0.60	≥ 0.95% level	7,288	6.5%	92.4%	1.1%	19.6%	36.0%

SaTScan	0.40	P1 (most likely) = 0.002	141	0.0%	100.0%	0.0%	14.9%	49.7%
		P2 (secondary) < 0.05	311	1.0%	98.4%	0.6%	9.6%	61.2%
	0.50	P1 (most likely) = 0.001	184	0.0%	100.0%	0.0%	12.5%	31.9%
		P2 (secondary) < 0.05	437	5.0%	93.8%	1.1%	16.9%	34.7%
	0.60	P1 (most likely) = 0.001	268	0.0%	99.6%	0.4%	11.6%	58.1%
		P2 (secondary) < 0.05	820	3.0%	96.3%	0.6%	13.2%	43.2%

## Discussion

The purpose of this study is to examine the spatial and population (e.g., socio-economic) characteristics of low birthweight using two different cluster estimation techniques. We compared the results of Kulldorff's Spatial Scan Statistic with the results of Rushton's Spatial filtering technique across increasing sizes of spatial filters (circle). Spatial filtering results did not show any particular area that was not statistically significant based on SaTScan. The high rates, which remain as the filter size increases to 0.4, 0.5 to 0.6 miles, respectively, indicate that these differences are less likely due to chance. The maternal characteristics of births within clusters differed considerably between the two methods. Progressively larger Spatial filters removed local spatial variability, which eventually produced an approximate uniform pattern of low birth weight.

As shown here both methods can be used for aggregated as well as point level data [21]. Although the two methods produced similar clusters there were some differences between them. Spatial filtering provided estimated risks for low birth weight incidence for each location in the map while SaTScan provides the statistical significance of the likely clusters after adjusting for multiple testing. Spatial filtering calculated risk estimates while using predetermined circle sizes defined either by geographical or "constant or near constant population size rather than constant geographic size" (p.2400) [21]. SaTScan on the other hand, uses circles of different sizes when searching over a grid [5]. The size of clusters identified by Spatial filtering in this example depended on the identified filter size, such as the radius of the circles.

As evidenced by this study, different filter sizes should be used to construct spatial filter maps when evaluating the two methods in terms of cluster size. Based on our findings, the Spatial filtering technique may provide many advantages over SaTScan. The resultant SaTScan detected cluster is a non-continuous circular shape, which often conceals spatial pattern – even though the actual geographic coordinates of each case and control are used through the Bernoulli method. Spatial filtering, on the other hand, treats low birth weight rates as a continuous spatial distribution.

The results from both softwares can easily be incorporated into GIS. SaTScan results provide the radius, latitude and longitude coordinates, and the P-value for the most likely clusters in a database or ASCII format. On the other hand, the Distance Mapping and Analysis Program (DMAP) produces morbidity rates using Spatial filters and tests for significance using Monte Carlo simulations. Its results are isarithmic maps that exhibit a continuous spatial distribution.

### Spatial filtering and DMAP

DMAP provides more consistent results if the total number of points is within the 20,000 range and when a finer grid size is used. It is preferable to break up the data to smaller units and time periods such as annual years to receive consistent results with Monte Carlo simulations. In our analysis, DMAP ran smoothly with a maximum data size of three years (42,394 births) and a maximum filter size of 0.6 mile. We did not receive consistent results when we used larger data sets and larger filter sizes. We believe this is related to the memory constraints of the current version of the DMAP software. When used with a smaller dataset, varying filter sizes of Spatial filtering showed cluster patterns very well and with consistent results.

### SaTScan and DMAP

With the current version of SatScan, we did not run into the problem of running large data sets. Specifically with our dataset, we ran SaTScan and got consistent results with more than 0.6 mile maximum cluster size. SaTScan can examine temporal effects much better than Spatial filtering method due to its ability to handle large data sets. With SaTScan, the use of discrete circular shapes represent only approximate locations of concentrated data counts [25]. Consequently, this technique does not provide useful information about the absolute proximity of clusters to point sources of contamination. However, some techniques have been proposed to improve SaTScan limitations [18,21].

## Conclusion

This study suggests that both estimation methods provide a useful way to characterize the spatial aspects of this birth outcome. The literature has strongly advocated the use of GIS in surveillance of the maternal environment and its impact on birth outcomes [12,25-33]. Yet, there are relatively few efforts to integrate and/or compare analytical techniques. SaTScan and Spatial filtering cluster estimation methods produced noticeably different results from the same individual level birth data. SaTScan clusters are likely to differ from Spatial filtering clusters in terms of population characteristics and geographic area within clusters. Using the two methods in conjunction could provide more detail about the population and spatial features contained with each type of cluster.

First, the two methods yielded many significant spatial clusters of low birthweight in Shelby County. We fully expected to find these clusters since the birth outcomes in this Midsouth area are historically among the nation's worst. The annual infant mortality rate in parts of Memphis frequently exceed 16 per 1,000 live births as opposed to about 10 per 1,000 live births in the state and about 7 per 1,000 live births nationally [34]. In 2002, 14% of children in Memphis were born prematurely as opposed to 12% nationally, while approximately 12% of children were born with low birth weight as opposed to 8% nationally [35]. Since 1996, about 40% of child deaths under the age of 18 resulted from prematurity [36].

Second, Spatial filtering clusters appear to cover much more geographic area han did SaTScan clusters. As we discussed earlier, this is likely due to the difference in basic assumptions between the two models. The two methods differ in their estimation of significance; SaTScan accounts for multiple testing of the highest likelihood of occurrence for all possible cluster locations and sizes while Spatial filtering does not. In addition, there may be somewhat of a "ceiling effect" with SaTScan. This maximum value ensures that the detected clusters, regardless of their location and size, are clusters detected without any pre-selection bias. The maximum allowed value of a spatial cluster does not mean that one has to pre-specify the size of a cluster before running an analysis. It simply means the largest allowed cluster would contain 50% of the at-risk population in the study area. This maximum value is reasonable because a cluster is expected to concentrate in certain areas of the study region. If a cluster covers most of the study area, then the location and size of the study area is no longer meaningful in that study area. Consequently, SaTScan clusters have an inherent but adjustable "cap" on cluster size whereas Spatial filtering is somewhat less limited. The tendency or capacity of Spatial filtering to yield clusters with considerably more geographic variability than SaTScan raises the issue of sample generalizability. Although not demonstrated in this study, the potential for greater variability of the characteristics of births within a Spatial filtering cluster may provide some analytic benefits.

Third, the maternal and familial characteristics of births contained within the two methods were remarkably alike. Additionally, changing the level of geographic scale resulted in very similar patterns between the two methods with respect to these characteristics. As the level of scale increased the sample became increasingly heterogeneous. We know that geographic scale is an important consideration in such investigations irrespective of the analytic method used [7]. This study found that clusters of low birthweight in Shelby County might extend into less impoverished, better educated, and more ethnically diverse communities.

We do not contend that either cluster estimation method is inherently superior. Instead this study underscores the need for an exploratory, integrative, and multi-scalar approach to assessing geographic patterns of disease, since different methods identify different patterns. First, both methods should be compared again using a Poisson approach. A temporal analysis of low birth weight could be conducted. Secondly, the two methods should be compared using different forms of chronic morbidity (i.e., congenital anomalies). Since other diseases differ in prevalence, population, and/or spatial characteristics, the results of the two methods might differ accordingly. Finally, the two methods should also be compared with respect to additional spatial characteristics. For example, the density or distribution of point sources of pollutants within either type of cluster could be examined on varying geographic scales.

## List of abbreviations

DMAP Distance Mapping and Analysis Program

GIS Geographic information systems

SaTScan Spatial Scan Statistic

ESRI Environmental System Research Institute

TIGER Topologically Integrated Geographic Encoding and Referencing System

## Authors' contributions

BW obtained data for this research. EO and BW conceptualized and conducted the analysis, and drafted the manuscript. EO directed development and interpretation of the spatial analysis. SK worked with EO on the methodology for imputation. MM worked with BW in the preparation and editing of the manuscript. All authors participated in the preparation and approved the final version of the manuscript.
